# Clinicopathological Characteristics and Mutational Landscape of *APC*, *HOXB13,* and *KRAS* among Rwandan Patients with Colorectal Cancer

**DOI:** 10.3390/cimb45050277

**Published:** 2023-05-16

**Authors:** Felix Manirakiza, Eric Rutaganda, Hidetaka Yamada, Yuji Iwashita, Belson Rugwizangoga, Benoit Seminega, Vincent Dusabejambo, Gervais Ntakirutimana, Deogratias Ruhangaza, Annette Uwineza, Kazuya Shinmura, Haruhiko Sugimura

**Affiliations:** 1 Department of Pathology, College of Medicine and Health Sciences, University of Rwanda, Kigali P.O. Box 3286, Rwanda; d19105@hama-med.ac.jp (F.M.);; 2 Department of Pathology, University Teaching Hospital of Kigali, Kigali P.O. Box 655, Rwanda; 3 Department of Tumor Pathology, Hamamatsu University School of Medicine, 1-20-1 Handayama, Higashi-Ku, Shizuoka 431-3192, Japan; h-yamada@hama-med.ac.jp (H.Y.); 07485223@hama-med.ac.jp (Y.I.);; 4 Department of Internal Medicine, University Teaching Hospital of Kigali, Kigali P.O. Box 655, Rwanda; 5 Department of Pathology, Butaro Hospital, Musanze P.O. Box 59, Rwanda; 6 Department of Biochemistry, Molecular Biology and Genetics, College of Medicine and Health Sciences, University of Rwanda, Kigali P.O. Box 3286, Rwanda; 7 Sasaki Institute Sasaki Foundation, 2-2 Kanda Surugadai, Chiyoda-Ku, Tokyo 101-0062, Japan

**Keywords:** colorectal cancer, genetics, *APC*, *KRAS*, *HOXB13*, mutation, polyposis syndrome, African, Rwanda

## Abstract

Cancer research in Rwanda is estimated to be less than 1% of the total African cancer research output with limited research on colorectal cancer (CRC). Rwandan patients with CRC are young, with more females being affected than males, and most patients present with advanced disease. Considering the paucity of oncological genetic studies in this population, we investigated the mutational status of CRC tissues, focusing on the *Adenomatous polyposis coli (APC)*, *Kirsten rat sarcoma* (*KRAS),* and *Homeobox B13 (HOXB13)* genes. Our aim was to determine whether there were any differences between Rwandan patients and other populations. To do so, we performed Sanger sequencing of the DNA extracted from formalin-fixed paraffin-embedded adenocarcinoma samples from 54 patients (mean age: 60 years). Most tumors were located in the rectum (83.3%), and 92.6% of the tumors were low-grade. Most patients (70.4%) reported never smoking, and 61.1% of patients had consumed alcohol. We identified 27 variants of *APC*, including 3 novel mutations (c.4310_4319delAAACACCTCC, c.4463_4470delinsA, and c.4506_4507delT). All three novel mutations are classified as deleterious by MutationTaster2021. We found four synonymous variants (c.330C>A, c.366C>T, c.513T>C, and c.735G>A) of *HOXB13*. For *KRAS*, we found six variants (Asp173, Gly13Asp, Gly12Ala, Gly12Asp, Gly12Val, and Gln61His), the last four of which are pathogenic. In conclusion, here we contribute new genetic variation data and provide clinicopathological information pertinent to CRC in Rwanda.

## 1. Introduction

Cancer in Africa is underinvestigated and underreported in the scientific literature [1,2,3], especially with respect to cancer genetics and genomics [4,5]. Available data highlight current and future increases in the incidence and mortality related to colorectal cancer (CRC) in African countries [3,6,7], and the Rwandan population is no exception [7]. 

Globally, CRC accounted for nearly 10% of cancer incidence and 9% of cancer deaths in 2020 and was ranked as the third-most deadly cancer [8,9]. 

Rwanda is an East African country with a population of 13,246,394 in 2022 [10]. Cancer-related research in this country is estimated to represent less than 1% of the total African cancer research output [1], and little research exists on cancer genetics in Rwanda [5,11,12,13,14,15]. 

Despite the paucity of CRC research, the few published data show that, in contrast to other parts of the world where CRC is more frequent in males and/or in older patients [8,16,17], in Rwanda, females are more affected than males [18,19] and many Rwandan CRC patients are young [18,19]. Moreover, most patients are diagnosed with advanced disease, a high number of patients are lost to follow-up, and the overall survival of patients with CRC is poor [18,19]. These effects may be due to the low density of healthcare personnel, limited infrastructure and specialized care, lack of awareness of CRC among the Rwandan population, and the unavailability of screening programs [20]. Furthermore, Awedew et al. [7] reported that Rwanda was among the African countries with the highest percentage change in CRC incidence cases between 2010 and 2019. This period coincides with an improvement in cancer diagnostic capacity within Rwanda as well as the emergence of specialized care. This includes the deployment of more surgeons in remote areas, the availability of chemotherapy, and other factors that may explain the increase in CRC incidence [21,22]. 

The role of heredity or genetic factors in CRC has been estimated to be approximately 35–40% [23,24]. Moreover, differences in genetic background and molecular characteristics associated with cancer between Africans and other races have also been reported [17,25]. Regarding CRC, differences in microsatellite stability status and somatic mutations have been documented [17,25,26,27]. In particular, the adenomatous polyposis coli (*APC*) and Kirsten rat sarcoma (*KRAS*) genes—both of which are among CRC driver genes [27,28]—have been reported to be more altered in patients of African ancestry [26]. 

*APC* is a multifunctional tumor suppressor gene [29] and is considered to be a key gatekeeper gene involved in CRC development [28,30]. Schell et al. [30] reported that *APC* may play a prognostic role in CRC and stated that routine clinical assessment of *APC* mutations together with *KRAS*, *TP53,* and *BRAF* may be used to predict CRC outcome. 

*KRAS* is the most frequently mutated oncogene in tumor tissues [31]. There are approved drugs targeting *KRAS*, while others are currently in clinical trials [31]. Recently, however, Zafra et al. [31], using a newly developed preclinical model, found that each allele-specific *KRAS* “mutation has distinct cellular consequences in vivo and carries differential sensitivity to targeted therapeutic agents”. Furthermore, a higher mutation rate in the *KRAS* gene has been reported in South African and African-American patients with CRC compared to Caucasians and/or other races [26,27,32,33]. 

Given the rising number of CRC cases in the Rwandan population, the young mean age of diagnosis, and the scarcity of oncogenetic studies in this population, we conducted this study to evaluate the mutational status in tumor tissues. We focused on the *APC* and *KRAS* genes, which are the most frequently mutated CRC driver genes. We also investigated the *HOXB13* gene, which has been reported to be underexpressed in distal colon cancer cases [34,35], but for which current data are conflicting regarding the association between its mutational status and CRC risk [34,36,37,38]. Our aim was, therefore, to elucidate any differences between the Rwandan population and other populations. As a result, in this paper, we describe the frequency of genetic variation in the *KRAS*, *HOXB13,* and *APC* genes, including novel mutations in *APC* in CRC tissues from Rwandan patients.

## 2. Materials and Methods

### 2.1. Ethical Approval and Consent to Participate

This study was approved by the Institutional Review Board (IRB) of the University of Rwanda College of Medicine and Health Sciences (Approval notice No. 048/CMHS IRB/2020, No. 113/CMHS IRB/2021, and No. 176/CMHS IRB/2022), the Ethics Committee of the University Teaching Hospital of Kigali (Ref.: EC/CHUK/2/064/2020) and the Ethics Committee of the Hamamatsu University School of Medicine (EC HUSM number: 20-011). All participants were informed of the purpose of the study, and they signed an informed consent form prior to participation in this study. The study was conducted in accordance with the Declaration of Helsinki.

### 2.2. Patients

From December 2020 to September 2022, we prospectively recruited consecutive cases of participants from patients attending colonoscopy services at the Department of Internal Medicine at the University Teaching Hospital of Kigali (CHUK, French acronym), Rwanda. Clinical data and self-reported information regarding the family history of cancer, alcohol, and tobacco use were recorded prospectively. Tissue processing and microscopic examination were performed at CHUK. The microscopic diagnosis was reconfirmed by a second pathologist at Hamamatsu University School of Medicine (HUSM) in Japan, and only patients with histologically confirmed CRC were included in this study. Initially, we graded cases according to the extent of glandular differentiation/formation using a three-tier grading system as follows: grade 1 (well-differentiated CRC, glandular formation >95%), grade 2 (moderately differentiated CRC, glandular formation 50–95%), and grade 3 (poorly differentiated CRC, glandular formation <50%) [39]. All CRC cases were then grouped into a two-tier grading system: low grade (i.e., including grade 1 and grade 2) and high grade (i.e., including grade 3), according to the recommendations of the fifth edition of the World Health Organization (WHO) classification of tumors of the digestive system [40]. During our study period, 148 consecutive patients underwent colonoscopic biopsy for suspected CRC. Of these, 129 (87.1%) signed an informed consent form to participate in our study. Here, 58 of 129 (44.9%) subjects had CRC, which was confirmed by biopsy, but 4 cases were excluded: one due to poor quality DNA, one due to a low concentration of extracted DNA, one case whose DNA could not be amplified by PCR, and one due to insufficient tissue for DNA extraction. The final number of cases analyzed and presented in this study was 54, and all cases included here were naïve to cancer therapy. 

### 2.3. DNA Extraction

Since our samples were collected prospectively, biopsy specimens were immediately fixed in a 10% neutral buffered formalin solution at the time of biopsy. After automated tissue processing, samples were embedded in paraffin. DNA extraction was performed before the tissues were six months old. For each case, the DNA was extracted from formalin-fixed paraffin-embedded (FFPE) tissue blocks containing at least 50% tumor cells. Extraction was performed using QIAamp DNA FFPE Advanced UNG Kits (Qiagen GmbH, Hilden, Germany), with all procedures following the manufacturer’s recommendations. The concentration and quality of extracted DNA were then measured using a Nanodrop^®^ 1000 spectrophotometer (ThermoFisher Scientific, Wilmington, CO, USA). As measured using the Nanodrop, the cases with a 260/280 ratio between 1.7 and 2.3 were included for further analysis.

### 2.4. DNA Amplification and Sanger Sequencing

For the *APC* gene, our sequencing targeted the mutation cluster region (MCR) [41,42,43,44] with flanking bases. The sequenced region was, therefore, GRCh 38: 112838850 to 112840618; this includes the MCR with flanking nucleotides. 

We also sequenced both exons (i.e., exons 1 and 2) of the HOXB13 gene [45]. Moreover, to cover a wide range of KRAS mutations in an understudied population—since there are no available genetic studies of Rwandans with CRC—we sequenced exons 2 to 4 of KRAS [46]. We also added a fragment of exon 5 extending from GRCh 38: 25209690 to 25209999 to include the hypervariable region (HVR) of this gene since mutations corresponding to the HVR are not included in some reports [47]. The boundaries of all sequenced exon segments are shown in Supplementary Table S1. Prior to sequencing, extracted DNA was amplified by PCR using HotStarTaq DNA Polymerase (Qiagen) in a 20 µL reaction volume. A list of primer sequences used to amplify the three genes is provided in Supplementary Table S1. The quality of PCR products was then assessed by electrophoresis on a 2% agarose gel. After amplification, PCR products were purified and sequenced as described in a previous study [48]. Briefly, PCR products were purified using ExoSAP-IT (ThermoFisher Scientific). The resulting purified products were directly Sanger sequenced in both directions using the BigDye^TM^ Terminator v3.1 Cycle Sequencing Kit (Applied Biosystems^TM^). This was performed in 20 µL reaction volumes, with all procedures performed according to the manufacturer’s recommendations. Base calling was performed using an Applied Biosystems 3130xl or 3500xL Genetic Analyzer (Applied Biosystems, Foster City, CA, USA).

### 2.5. TA Cloning and Plasmid Sequence Analysis for Insertion/Deletion Cases

TA cloning was performed to assess cases with insertion/deletion mutations. Note that two separate cloning experiments (i.e., including two different transformations, with two PCR products, obtained in separate reactions) were used to eliminate possible polymerase-induced errors. 

#### 2.5.1. Ligation Reaction Using pGEM^®^-T Easy Vector System 

First, PCR products were purified using QIAquick^®^ PCR Purification Kits (Qiagen GmbH, Hilden, Germany). Next, we constructed plasmids by ligating purified PCR products to a pGEM^®^-T Easy Vector System (Promega Corporation, Madison, WI, USA) with all procedures following the manufacturer’s protocol.

#### 2.5.2. Transformation Using Highly Competent DH5α *E. coli* Cells 

COMPETENT High DH5α competent *E. coli* cells (Toyobo Corporate, Osaka, Japan) were then transformed with the constructed plasmid. All procedures were performed according to Promega’s protocol, except that we used DH5α *E. coli* cells (Toyobo). Furthermore, we prepared fresh X-Gal/IPTG Luria-Bertani (LB) agar plates containing ampicillin at a final concentration of 100 µg/µL according to the protocol of ThermoFisher Scientific (i.e., to each 25 mL of LB agar, we added 40 µL of X-Gal solution (20 mg/mL) and 40 µL of IPTG (100 mM); then, the plates were allowed to dry under UV light at room temperature before use).

#### 2.5.3. Screening for Transformants followed by Sanger Sequencing of Positive Subclones 

Transformed cells were grown overnight on X-gal/IPTG LB agar plates described above, allowing the selection of blue/white colonies. White colonies (i.e., eight subclones) were then selected [48] and each cultured in 50 µL of liquid LB medium containing ampicillin at a final concentration of 100 µg/µL for 1 h and 30 min at 37 °C. The resulting cultures were centrifuged, and 1 µL of the lower phase was used for PCR in a 20 µL volume using HotStartTaq (Qiagen) polymerase enzyme and pGEM Easy V-2930F (5′-AGG CGA TTA AGT TGG GTA ACG-3′) and pGEM Easy V-134R (5′-CAA GCT ATG CAT CCA ACG C-3′) primers. The resulting PCR products were then purified and sequenced as described above.

### 2.6. Mutation Detection, Annotation, and In Silico Analysis

ABI sequences were aligned to the respective genomic sequences for each gene (i.e., NC_000005.10 for *APC*, NC_000017.11 for *HOXB13*, and NC_000012.12 for *KRAS*) using both UniproUGENE version 45 [49] and GENETYX^®^ version 14.1.0 (Genetyx Corporation, Tokyo, Japan).

Known variants were then annotated using the dbSNP (build 156) database as per the recommendations of the Human Genome Variation Society (HGVS) [50]. Novel variants were analyzed using MutationTaster2021 [51] in silico tools to predict the consequences of the resulting proteins. Each detected mutation was confirmed by two independent experiments to exclude false mutations. We then consulted major databases (i.e., ALFA, ClinVar, dbSNP, VariSome, 1000 Genome Project, TOPMed, and COSMIC) before concluding that a mutation was novel. Finally, since we used tumor tissue, we could not determine whether mutations were purely somatic or were also present in the germline. 

## 3. Results

### 3.1. Clinicopathological Characteristics of Patients

In this study, we analyzed data from 54 patients with a mean age of 60 ± 13 years (range 31–89 years). Our dataset included 34 females (63%) and 20 males (37%). We found that tumors were mainly located in the rectum (45 cases, 83.3%) or colon (8 cases, 14.8%) with only one case of anorectal tumor (1.9%). All cases were biopsies and histologically confirmed as adenocarcinoma. In total, 50 cases (92.6%) were low-grade and 4 cases (7.4%) were of high-grade. Most patients (38 cases, 70.4%) reported that they had never smoked, while 33 patients (61.1%) reported that they had consumed alcohol. Detailed clinical data are presented in Table 1. 

### 3.2. Characteristics of APC Genetic Variants

**Figure 1:**
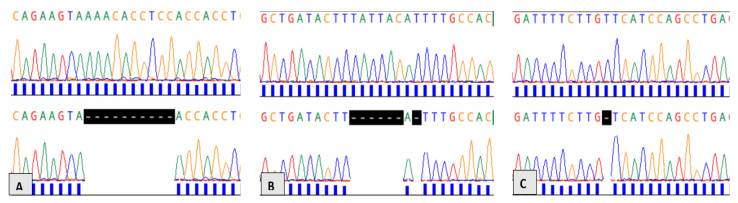
Novel mutations in the *APC* gene. (**A**) mutation c.4310_4319delAAACACCTCC, (**B**) mutation c.4463_4470delinsA and (**C**) mutation c.4506-4507delT. Upper electropherograms correspond to the wild-type *APC* sequence and lower electropherograms correspond to mutations at the same positions.

We found 27 different genetic variants (Table 2). These included 3 novel genetic mutations (Figure 1), 23 previously reported mutations, and 1 single-nucleotide polymorphism (SNP), rs41115. This SNP has a minimal allele frequency (MAF) of >50% in major databases. Excluding rs4115, 27 (27/54; 50%) cases were identified to have at least 1 mutation. Here, 1 case had 3 mutations, and 4 cases had 2 different mutations each, yielding a total of 33 mutations. Recurrent mutations included rs74380081 (4/54), rs386833391 (3/54), rs121913224 (2/54), rs139387758 (2/54), and rs587783031 (2/54). Further, 19 of 27 (70.3%) mutations were nonsense or frameshift mutations that led to a premature stop codon and are, therefore, classified as pathogenic or deleterious variants. Seven mutations are benign or likely benign (7/27; 25.9%) and one (1/27, 3.70%) variant was of uncertain significance. 

### 3.3. Characteristics of HOXB13 Genetic Variants

We identified four different genetic variants: rs33993186 (c.330C>A; p.Pro110=), rs8556 (c.366C>T; p.Ser122=), rs9900627 (c.513T>C; p.Ser171=), and rs138675188 (c.735G>A; p.Lys245=), all of which are synonymous variants and were classified as benign or likely benign (Table 3). However, the *HOXB13* Ter285Lys variant, which has recently been described in the West African population [52], and the most commonly studied *HOXB13* Gly84Glu variant were both absent in our study participants. 

### 3.4. Characteristics of Genetic Variants in the KRAS Gene

Next, analysis of the *KRAS* gene identified six different genetic variants including four pathogenic variants (i.e., Gly12Ala, Gly12Asp, Gly12Val, and Gln61His), one variant with a conflicting interpretation of pathogenicity (Gly13Asp), and one SNP (NM_004985.5: c.519T>C) that leads to a synonymous amino acid (Asp173=). Detailed information regarding these mutations is provided in Table 4.

## 4. Discussion

CRC is underinvestigated in the Rwandan population. To our knowledge, only two papers [18,53] and one abstract [19] reporting on CRC in Rwanda have been published in international peer-reviewed journals within the last 10 years. In contrast to global data where CRC is reported to be more common in males than in females [9], in this study, we found that CRC was more common in females than in males (i.e., 63.0% versus 37.0%). Our findings also report a higher rate of female incidence than the 52.5% reported by Uwamariya et al. [18] and the 52.2% reported by Fadelu et al. [19].

Most tumors were located in the rectum (83.3%), which may be due to the fact that rectal tumors are more symptomatic (i.e., they display tenesmus, pain during defecation, bleeding, and blood in the stool, among other symptoms) than colon tumors. Therefore, patients with rectal tumors are more likely to seek medical care relative to patients with colon tumors. In addition, screening programs for CRC are not available in our settings [20]. As a result, most patients with colon tumors present with advanced stages of cancer and signs of obstruction. They, therefore, immediately undergo resection to relieve the obstruction without undergoing colonoscopy. This speculation may be supported by the fact that Uwamariya et al. [18] found that rectal cancer accounted for 40% of all CRC cases in a study conducted at the same institution as ours that used both biopsy and resection specimens.

We also found that 35.2% (19/54) of our research participants reported that they had no information regarding a family history of cancer. This is different from missing information that was not recorded, which accounted for 3.7% (2/54 cases) in this study. In addition, 61.1% of CRC patients reported that they had “no family history of cancer.” Therefore, none of the patients in this study reported having a family history of cancer. We speculate that self-reported data regarding family history of cancer may not necessarily mean that cancers were absent, especially because of limited opportunities to receive a cancer diagnosis, treatment, and cancer registration in the past [20]. Therefore, data on family history of cancer should be interpreted with caution, and prospective studies with good documentation regarding cancer-related information are recommended. 

### 4.1. New Mutations in the APC Gene

The *APC* gene is a key gatekeeper gene involved in the development of CRC [28,30]. Germline mutations in *APC* are also key factors in familial adenomatous polyposis syndrome. This syndrome is quasi-nonexistent in African populations, with only a few cases having ever been reported in the literature [54,55,56,57,58,59,60,61,62,63]. Mutations in the *APC* gene in CRC have been reported in more than 50% of cases [44]. Moreover, >60% of *APC* mutations are located in the MCR [44,64,65,66]. In this study, we limited our genetic assessment to a region including the MCR with ±300 bases flanking either side. In total, we detected 27 genetic variants or mutations, 24 of which have been previously documented in the dbSNP, ClinVar, and/or COSMIC databases. 

Three mutations, i.e., c.4310_4319delAAACACCTCC: p.Lys1437Asnfs*33, c.4463_4470delinsA: p.Leu1488Tyrfs*17, and c.4506_4507delT: p.Ser1503Hisfs*4 have, to our knowledge, not yet been reported in the literature or major genetic variation databases. All these mutations are frameshift mutations that are predicted to cause a premature termination codon and therefore produce a truncated protein. Using in silico bioinformatics tools, each of these genetic variants is predicted to be deleterious and to cause nonsense-mediated mRNA decay.

### 4.2. Variants in the HOXB13 Gene

We identified four different genetic variants: rs33993186, rs8556, rs9900627, and rs13865188, all of which are synonymous and classified as benign or likely benign. Silva et al. [34] only reported the rs8556 and rs9900627 variants in CRC samples from patients treated at the Portuguese Institute of Oncology-Porto. However, in our study, we found cases containing the rs33993186 and rs138675188 variants. The rs33993186 (g.487282264G>T) variant and the rs138675188 (g.48726910C>T) variant appear to be more than two times more common in Africans than in the global population as currently reported in dbSNP build 156. 

The *HOXB13* Gly84Glu variant found mostly in the Europeans [67,68], the Gly132Glu and Gly135Glu mutations described in the Asians [69,70], and the *HOXB13* Ter285Lys variant found mostly in the West African populations [52] were all absent in our study participants. 

### 4.3. KRAS Missense Mutations

Five of the six *KRAS* variants identified in this study are missense mutations. These mutations included the pathogenic mutations Gly12Asp, Gly12Ala, Gly12Val, and Gln61His as well as the variant Gly13D, which had conflicting interpretations of pathogenicity. These five missense mutations are the most commonly reported *KRAS* mutations and were present in 22 of 54 samples (40.7%) analyzed here. Ben Salah et al. [71] reported a *KRAS* mutation rate of 55.7%, while Marbun et al. [72] reported a rate of 64%. In other studies, either Gly12Asp [73,74] or Gly12Val [71,75] were the most common *KRAS* mutations. However, in our study, Gly13Asp was the most recurrent mutation, followed by Gly12Val. *KRAS* Gly12Asp, which has been reported to be associated with the best prognosis [73], was present in 2/54 of our patients, and Gly12Cys, which is associated with a poor prognosis [73], was not identified in any patients in our study. 

### 4.4. Smoking, Alcohol Consumption, Diet, and Other Environmental Risk Factors for CRC

A total of 20% (11/54) of our study population reported ever having smoked and 61.1% (33/54) reported ever having consumed alcohol. Both proportions are higher than the 6.9% (for smokers) and 14.7% (for drinkers) reported by Wismayer et al. [76] in the Ugandan population neighboring Rwanda. In addition, Wismayer et al. [76] found that former smokers and current and former drinkers had a higher risk of developing CRC. 

It has been reported that a high intake of red meat, processed meat, sweetened beverages, a diet low in fiber, and a low intake of dairy products [77,78,79,80] are factors associated with an increased risk of developing CRC. Although we did not assess the diet of our study participants, it is worth noting that the Rwandans rarely consume meat, fish, fruit, and dairy products, but their diet may be rich in starchy foods [81,82,83]. 

### 4.5. Limitations

Our study has some limitations. We only used tumor tissues. Therefore, we could not confirm whether genetic variations and/or mutations were purely somatic or also present in the germline. By using Sanger sequencing, we were only able to investigate a small number of cases and genes. Furthermore, with respect to the *APC* gene, our analysis was limited to only a small portion of this gene. In addition, we did not investigate any genes associated with Lynch syndrome, nor did we determine the chromosomal stability status of our study participants. Further studies using next-generation sequencing techniques are recommended to investigate a large number of genes in a larger pool of cases. Our clinical data did not include the cancer stage; therefore, we provided limited pathological characteristics of our patients. Furthermore, information on smoking, alcohol consumption, and family history of cancer was self-reported, and we were not able to make an objective estimate of the amounts consumed. Therefore, we could not estimate an association between these factors and the presence or absence of a particular mutation. Finally, we were not able to perform immunohistochemical staining or functional studies to further investigate the effect of the described mutations on the expression levels of corresponding proteins and/or to confirm the in silico predicted mutational effect. 

## 5. Conclusions

In this study, we contribute new genetic variation data and clinicopathological information relevant to CRC patients in Rwanda. This genetic information does not yet reflect specific environmental (i.e., dietary and other) components. However, additional studies are required to improve the quality of scientific data characterizing CRC cases in the Rwandan population and improve evidence-based management of this disease. The genetic data provided in this paper also represent a valuable resource for the study of CRC in understudied populations. 

## Figures and Tables

**Table 1:** Clinical characteristics of all included cases.

Case No	Age at Diagnosis	Sex	Site	Grade	Ever Smoker (Self-Reported)	Ever Alcohol Intake (Self-Reported)	Family History of Cancer (Self-Reported)	APC Mutation ^#^	KRAS Mutation
1	49	F	Rectum	Grade 2	No	Yes	No		
2	59	F	Rectum	Grade 2	No	No	No	Yes	
3	31	M	Rectum	Grade 1	No	Yes	No	Yes	Yes
4	83	F	Anorectum	Grade 2	NA	NA	No information	Yes ^2^	Yes
5	47	F	Rectum	Grade 3	No	Yes	No	Yes	
6	50	M	Rectum	Grade 2	No	Yes	No		
7	89	F	Rectum	Grade 1	Yes	Yes	No		
8	61	F	Rectum	Grade 1	NA	NA	No	Yes ^3^	Yes
9	66	F	Colon	Grade 3	No	No	No		Yes
10	32	F	Rectum	Grade 3	No	Yes	No	Yes	Yes
11	76	F	Rectum	Grade 3	No	Yes	No information		
12	51	F	Rectum	Grade 1	No	No	No information		
13	81	M	Rectum	Grade 2	Yes	Yes	No	Yes	
14	49	F	Rectum	Grade 2	No	No	No	Yes	
15	72	M	Rectum	Grade 1	No	Yes	No		
16	63	F	Rectum	Grade 2	No	Yes	No		Yes
17	59	F	Rectum	Grade 2	No	Yes	No	Yes	Yes
18	61	F	Colon	Grade 1	No	No	No	Yes ^2^	Yes
19	68	M	Rectum	Grade 2	No	Yes	No information	Yes	Yes
20	59	F	Rectum	Grade 1	No	Yes	No information	Yes	
21	53	M	Colon	Grade 2	No	Yes	No	Yes	Yes
22	61	M	Rectum	Grade 1	Yes	Yes	No	Yes	Yes
23	59	M	Rectum	Grade 2	No	Yes	No		Yes
24	64	F	Rectum	Grade 1	No	Yes	No information	Yes ^2^	
25	63	M	Colon	Grade 1	No	Yes	No		Yes
26	49	M	Colon	Grade 1	No	Yes	No information	Yes	Yes
27	56	M	Rectum	Grade 1	Yes	Yes	No information		Yes
28	73	F	Rectum	Grade 2	No	Yes	No information		
29	77	F	Rectum	Grade 2	Yes	Yes	No information	Yes	
30	73	M	Rectum	Grade 2	Yes	Yes	No		Yes
31	62	M	Rectum	Grade 2	NA	Yes	No	Yes	Yes
32	79	F	Rectum	Grade 2	No	Yes	No information		
33	50	F	Rectum	Grade 2	No	No	No		
34	67	F	Rectum	Grade 1	No	No	No	Yes ^2^	Yes
35	65	F	Rectum	Grade 2	No	Yes	No	Yes	
36	45	M	Rectum	Grade 1	No	Yes	No information	Yes	
37	40	M	Rectum	Grade 2	NA	NA	NA		
38	51	M	Rectum	Grade 2	No	No	No	Yes	Yes
39	57	F	Rectum	Grade 1	Yes	No	No information		
40	63	F	Rectum	Grade 2	Yes	Yes	No information		
41	78	F	Rectum	Grade 2	Yes	Yes	No information		Yes
42	38	M	Rectum	Grade 1	No	No	No		
43	45	F	Rectum	Grade 1	No	No	No		
44	61	M	Rectum	Grade 2	No	No	No		
45	63	F	Colon	Grade 2	No	Yes	No	Yes	
46	75	F	Rectum	Grade 1	Yes	Yes	No information		Yes
47	54	F	Rectum	Grade 2	Yes	Yes	No information	Yes	
48	36	M	Rectum	Grade 2	No	No	No information		Yes
49	65	M	Rectum	Grade 1	No	No	No	Yes	
50	68	F	Colon	Grade 2	No	No	No		
51	52	F	Rectum	Grade 2	NA	NA	NA		
52	64	F	Rectum	Grade 2	No	Yes	No		
53	74	F	Rectum	Grade 2	No	Yes	No	Yes	
54	59	F	Colon	Grade 1	No	NA	No information	Yes	

NA: not available, ^#^: Superscript numbers in this column indicate the number of *APC* mutations found in the corresponding case. Variants at rs41115 are not included.

**Table 2:** Details of APC variants in Rwandan patients with colorectal cancer.

No	dbSNP ID	GRCh38 Coordinate (NC-000005.10)	NM_000038.6	Protein Change (NP_000029.2)	Codon Change	Clinical Significance ^$^	Number of Cases with Mutation (N = 54)	MAF ALFA/Global	MAF ALFA/ African	MAF 1000G/ Global	MAF 1000G/ African
1	rs121913331	g.112838934C>T	c.3340C>T	p.Arg1114Ter	CGA>TGA	Pathogenic	1	NA	NA	NA	NA
2	rs386833391	g.112839054-112839056delGAA	c.3460_3462delGAA	p.Glu1157del	NA*	Benign	3	0.00007	0.0009	0.0048	0.0174
3	rs138933660	g.112839244A>C	c.3650A>C	p.Asn1217Thr	AAT>ACT	Likely benign/US	1	0.000065	0.000014	0.001	0.0038
4	rs74380081	g.112839326A>G	c.3732A>G	p.Glu1244=	CAA>CAG	Benign	4	0.00246	0.0282	0.0142	0.0537
5	NA	g.112839451_112839453dupA	c.3859dupA	p.Ile1287Asnfs*4	NA*	Deleterious (in silico)	1	NA	NA	NA	NA
6	rs1064794229	g.112839514_112839518delTAAAA	c.3920_3924delTAAAA	p.Ile1307Argfs*6	NA*	Pathogenic	1	NA	NA	NA	NA
7	rs121913224	g.112839515_112839525delAAAGA	c.3927_3931delAAAGA	p.Glu1309Aspfs*4	NA*	Pathogenic	2	0.0001	Zero	NA	NA
8	NA	g.112839534delA	c.3940delA	p.Arg1314Glyfs*7	NA*	Deleterious (in silico)	1	NA	NA	NA	NA
9	rs1057517558	g.112839549_112839550delC	c.3956delC	p.Pro1319Leufs*2	NA*	Pathogenic	1	NA	NA	NA	NA
10	rs1554085480	g.112839661C>A	c.4067C>A	p.Ser1356Ter	TCA>TAA	Pathogenic	1	NA	NA	NA	NA
11	rs121913328	g.112839693C>T	c.4099C>T	p.Glu1367Ter	CAG>TAG	Pathogenic	1	NA	NA	NA	NA
12	NA	g.112839702A>T	c.4108A>T	p.Lys1370Ter	AAA>TAA	Pathogenic	1	NA	NA	NA	NA
13	rs1580645082	g.112839783G>T	c.4189G>T	p.Glu1397Ter	GAG>TAG	Pathogenic	1	NA	NA	NA	NA
14	rs587782518	g.112839810C>T	c.4216C>T	p.Gln1406Ter	CAG>TAG	Pathogenic	1	NA	NA	NA	NA
**15**	**NA**	**g.112839904_112839913delAAACACCTCC**	**c.4310_4319delAAACACCTCC**	p.Lys1437Asnfs*33	NA*	**Deleterious (in silico)**	**1**	**NA**	**NA**	**NA**	**NA**
16	rs111866410	g.112839954A>G	c.4360A>G	p.Lys1454Glu	AAA>GAA	Benign	1	0.000392	0.0099	0.0008	0.0023
17	rs387906234	g.112839979_112839988delAG	c.4393_4394delAG	p.Ser1465Trpfs*3	NA*	Pathogenic	1	NA	NA	NA	NA
18	Rs387906234	g.112839979_112839988dupAG	c.4393_4394dupAG	p.Ser1465Argfs*9	NA*	Deleterious (in silico)	1	NA	NA	NA	NA
19	NA	g.112840012A>G	c.4418A>G	p.Asn1473Ser	AAT>AGT	US	1	NA	NA	NA	NA
20	rs139387758	g.112840014G>A	c.4420G>A	p.Ala1474Thr	GCT>ACT	Benign	2	0.000326	0.0105	0.0026	0.0098
**21**	**NA**	**g.112840057_112840064delinsA**	**c.4463_4470delinsA**	p.Leu1488Tyrfs*17	NA*	**Deleterious (in silico)**	**1**	**NA**	**NA**	**NA**	**NA**
22	rs41115	g.112840073G>A	c.4479G>A	p.Thr1493=	ACG>ACA	Benign	28	0.61	0.53	0.665	0.517
**23**	**NA**	**g.112840100_112840101delT**	**c.4506_4507delT**	p.Ser1503Hisfs*4	NA*	**Deleterious (in silico)**	**1**	**NA**	**NA**	**NA**	**NA**
24	rs587783031	g.112840255_112840260delA	c.4666delA	p.Thr1556Leufs*9	NA*	Pathogenic	1	NA	NA	NA	NA
25	rs587783031	g.112840255_112840260dupA	c.4666dup	p.Thr1556Asnfs*3	NA*	Pathogenic	2	NA	NA	NA	NA
26	rs35634377	g.112840487T>C	c.4893T>C	p.Ser1631=	AGT>AGC	Benign/Likely benign	1	0.00098	0.0104	0.0008	0.0023
27	rs1554086285	g.112840496_112840500dupG	c.4906dupG	p.Asp1636Glyfs*2	NA*	Pathogenic	1	NA	NA	NA	NA

N: total number of cases, NA: not available, NA*: not applicable, US: uncertain significance, ^$^: We used clinical significance values reported in ClinVar except where we specified that these values were determined using in silico tools (i.e., using MutationTaster2021). Novel mutations are shown in bold font.

**Table 3:** Details of *HOXB13* variants in Rwandan patients with colorectal cancer.

No	dbSNP_ID	GRCh38 Coordinate	NM_006361.6	Protein Change NP_006352.2	Codon Change	Clinical Significance ^$^	Number of Cases with Mutation (N = 54)	MAF ALFA/Global	MAF ALFA/ African
1	rs33993186	g.48728264G>T	c.330C>A	p.Pro110=	CCC>CCA	benign	6	0.00403	0.0220
2	rs8556	g.48728228G>A	c.366C>T	p.Ser122=	AGC>AGT	benign	26	0.131	0.172
3	rs9900627	g.48728081A>G	c.513T>C	p.Ser171=	TCT>TCC	benign	20	0.104	0.117
4	rs138675188	g.48726910C>T	c.735G>A	p.Lys245=	AAG>AAA	Likely benign	1	0.00055	0.0024

N: total number of cases, ^$^: Clinical significance values reported in ClinVar.

**Table 4:** Details of *KRAS* variants in Rwandan patients with colorectal cancer.

No	dbSNP_ID	GRCh38 Coordinate	NM_004985.5	Protein Change (NP_004976.2)	Codon Change	Clinical Significance ^$^	Number of Cases with Mutation (N = 54)	MAF ALFA/ Global	MAF ALFA/ African
1	rs121913529	g.25245350C>T	c.35G>A	p.Gln12Asp	GGT>GAT	Pathogenic	2	0.00001	Zero
2	rs121913529	g.25245350C>G	c.35G>C	p.Gln12Ala	GGT>GCT	Pathogenic/ likely pathogenic	1	NA	NA
3	rs121913529	g.25245350C>A	c.35G>T	p.Gln12Val	GGT>GTT	Pathogenic	7	NA	NA
4	rs112445441	g.25245347C>T	c.38G>A	p.Gly13Asp	GGC>GAC	C.I	11	NA	NA
5	rs17851045	g.25227341T>G	c.183A>C	p.Gln61His	CAA>CAC	Pathogenic/ likely pathogenic	1	NA	NA
6	rs1137282	g.25209843A>G	c.519T>C	p.Asp173=	GAT>GAC	Benign	19	0.213945	0.1763

^$^: Clinical significance values reported in ClinVar, C.I: Conflicting interpretation of pathogenicity, N: total number of cases, NA: not available.

## Data Availability

The data presented in this study are available on request from the corresponding author.

## Supplementary Materials

The following supporting information is submitted as supplementary material and can be downloaded at: https://www.mdpi.com/article/10.3390/cimb45050277/s1. Table S1: List of primer pairs for PCR amplification.

## Abbreviations

ALFA	Allele frequency aggregator
*APC*	Adenomatous polyposis coli
CHUK	University Teaching Hospital of Kigali
CMHS	College of Medicine and health sciences
COSMIC	Catalogue of somatic mutation in cancer
DNA	Deoxyribonucleic acid
*HOXB13*	Homeobox B13
IRB	Institution review board
*KRAS*	Kirsten rat sarcoma
LMICs	Low- and middle-income countries
SNP	Single-nucleotide polymorphism
WHO	World health organization
